# Synchronous detection of *Burkholderia pseudomallei* and its ceftazidime resistance mutation based on RNase-HII hydrolysis combined with lateral flow strip assay

**DOI:** 10.1128/spectrum.01125-23

**Published:** 2023-10-10

**Authors:** Juan Yao, Zhang Zhang, Shen Tian, Nini Luo, Jun Tan, Yue Zhang, Shuo Gu, Qianfeng Xia

**Affiliations:** 1 Key Laboratory of Tropical Translational Medicine of Ministry of Education, NHC Key Laboratory of Tropical Disease Control, School of Tropical Medicine, Hainan Medical University, Haikou, Hainan, China; 2 Nanobiosensing and Microfluidic Point-of-Care Testing Key Laboratory of LuZhou, Luzhou, Sichuan, China; 3 Department of Neurosurgery, Neurology Center, The First Affiliated Hospital of Hainan Medical University, Haikou, Hainan, China; JMI Laboratories, North Liberty, Iowa, USA

**Keywords:** melioidosis, *Burkholderia pseudomallei*, ceftazidime resistance mutation, rhPCR, lateral chromatographic flow strip, universal probes

## Abstract

Melioidosis is a severe and often fatal infectious disease caused by *Burkholderia pseudomallei*, which poses significant challenges due to its high mortality rate and frequent misdiagnosis. To effectively treat infected patients, especially in epidemic regions like Hainan with a high resistance rate to ceftazidime (CAZ), early diagnosis and resistance testing are critical. Here, we designed a strategy that combines RNase-HII-dependent PCR with lateral flow strip assay (LFSA) for simultaneous detection of *B. pseudomallei* and its CAZ resistance mutation. In this strategy, we utilized rhPCR technology to design specific primers with a C3-spacer modification. These primers target a 115-base pair region within the ORF2 of the *B. pseudomallei* type III secretion system gene cluster, as well as the P174L mutation in the *penA* gene, which is a major mutation associated with CAZ resistance in Hainan. The primers contain complementary RNA bases that can be hydrolyzed only in the presence of thermostable RNase-HII enzymes, facilitating subsequent PCR reactions. Additionally, we introduced two pairs of universal probes carrying fluorescein isothiocyanate (FITC) and biotin, as well as digoxin and biotin. The sequences of these universal probes align with specific regions of the primers, resulting in target products labeled with different tags. The resulting amplification products are then subjected to LFSA, where the presence of specific markers is visually interpreted based on color development. These markers flow to test line 1 (T1) and are captured by a fixed anti-FITC antibody, generating a red line that indicates the presence of *B. pseudomallei*. Similarly, the occurrence of a red line on test line 2 (T2), which is fixed with anti-digoxin antibodies, indicates the presence of the P174L mutation in *B. pseudomallei*. This protocol serves as an excellent tool for the rapid diagnosis of melioidosis and provides a basis for selecting appropriate antibiotic medication. It has the potential to revolutionize the diagnosis of melioidosis, particularly in resource-limited settings. Furthermore, this technique holds promise as a versatile tool for disease diagnosis in general.

**IMPORTANCE:**

This study focused on the development of a reaction system using rhPCR to amplify a specific gene, ORF2, of *B. pseudomallei* and to identify the P174L mutation associated with increased drug resistance to ceftazidime (CAZ). The system incorporated universal primer probes and a simple temperature cycle reaction. The amplified products were then analyzed using lateral flow strip assay (LFSA) for strain identification and mutation interpretation. The developed system provides a reliable basis for diagnosing melioidosis and selecting appropriate drugs. Its potential impact is particularly significant in resource-limited settings where access to advanced diagnostic techniques is limited. This platform stands out for its simplicity, convenience, sensitivity, specificity, and portability. It shows promise as a point-of-care testing method for detecting single nucleotide polymorphism in genes associated with other diseases. By leveraging the advantages of this platform, researchers and healthcare professionals can potentially expand its use beyond melioidosis and apply it to the rapid detection of genetic variations in other disease-related genes.

## INTRODUCTION

*Burkholderia pseudomallei* (*B. pseudomallei*) is a medium-sized Gram-negative cocci bacterium that belongs to the Burkholderia genus. It is primarily found in the rhizosphere, wet soil, surface water, and ground water (1). It is an opportunistic pathogen responsible for causing melioidosis, a disease that can be contracted through inhalation or when damaged skin comes into contact with contaminated water or soil. Melioidosis can lead to various health complications, including pneumonia, lung abscess, skin and soft tissue infections, and sepsis (2, 3). In severe cases, death can occur within 48 hours of the onset of symptoms (4). Research indicates that melioidosis affects approximately 165,000 individuals worldwide and causes around 89,000 deaths each year (5). The disease is endemic in tropical and subtropical regions, primarily occurring in parts of Southeast Asia, the Indian subcontinent, and northern Australia (5, 6). In China, Hainan is recognized as one of the traditionally affected areas by melioidosis, and the presence of the pathogen has been detected along the coastline (7, 8). Factors such as global climate change, increased transportation convenience, and frequent economic, trade, and cultural exchanges have contributed to the rising infection rates and expanding geographic range of melioidosis over the years (5). However, due to limitations in diagnostics and a lack of awareness, melioidosis is often underestimated in many tropical countries where the disease is endemic or expected to become endemic.

Early and effective antimicrobial therapy, along with proper treatment duration and rational drug selection, is crucial for the successful management of melioidosis. The treatment of melioidosis typically involves two stages: an intensive treatment phase, aimed at rapidly killing or inhibiting *B. pseudomallei* in patients, followed by an eradication treatment phase, which aims to eliminate residual bacteria and minimize the risk of infection recurrence (9). Given that standard therapies for sepsis are ineffective against *B. pseudomallei* infection, early and accurate diagnosis is particularly important for the appropriate selection of antimicrobial drugs and effective treatment of melioidosis (10). One major challenge in the treatment process is the intrinsic drug resistance exhibited by *B. pseudomallei*, which encompasses nearly all known resistance mechanisms such as enzymatic inactivation, efflux pumps, reduced permeability, biofilm formation, genetic mutation, and acquired resistance genes (11). Although ceftazidime (CAZ) and meropenem are commonly used as first-line drugs, clinical strains of *B. pseudomallei* can display heterogeneous drug resistance, leading to prolonged treatment duration and high recurrence rates (12, 13). Hainan province in China, with a high incidence of melioidosis, has experienced a significant increase in resistance rates, likely due to the rising number of melioidosis cases and the misuse and overuse of antibiotics. A retrospective study of 164 melioidosis cases over a 13-year period in Hainan province showed a remarkably high CAZ resistance rate of 12.8%, surpassing global rates (14). *B. pseudomallei*-specific resistance arises from genetic alterations in the chromosome. Research has identified *penA*, the gene encoding class A β-lactamase, as a key player in the resistance mechanism of *B. pseudomallei*. Mutations within *penA* can result in the inactivation of susceptible antibiotics through hydrolysis or trapping mechanisms (15, 16). Clinically relevant resistance to CAZ is primarily caused by mutations in *penA*. Primary resistance of *B. pseudomallei* to CAZ is relatively rare, and acquired resistance is associated with receiving multiple courses or extended treatment with CAZ. The main mechanisms include the following: (i) mutations in the promoter region leading to increased *penA* expression, (ii) gene dosage amplification events in the chromosomal region of the *penA*, and (iii) point mutations in the *penA*, including P167S, C69Y, and D240G (12, 13, 17). Whole-genome analysis of *B. pseudomallei* strains isolated from clinical cases in Hainan identified a total of 32-point mutations and 7 indels in *penA*, including notable mutations such as I139M, P145L, T147A, and T264A (18). In addition, it has been confirmed that the P174L mutation in *penA* can cause enhanced CAZ resistance by expanding the space in a conserved structure called the omega loop, which in turn increased flexibility at the active site (19). The previous study of our group (under review) also showed that this locus is also the main mechanism of CAZ resistance in *B. pseudomallei* in Hainan. The presence of these mutation sites severely limits the effectiveness of CAZ, underscoring the importance of timely and accurate identification of these mutations to enable doctors to adjust treatment strategies promptly.

Currently, the gold standard for diagnosing melioidosis relies primarily on the bacterial culture of clinical isolates, followed by serology, mass spectrometry, and other identification methods (20, 21). However, these methods often have limitations such as low sensitivity, time-consuming procedures, complexity, and high requirements for technical expertise and equipment (22). Although quantitative real-time PCR technology has reduced identification time significantly, it still involves complex nucleic acid extraction steps, specialized equipment, and strict laboratory safety protocols and has a sensitivity of only around 60%–70% (23, 24). Other techniques based on antigen-antibody reactions such as latex agglutination, immunofluorescence analysis (LAA), lateral flow immunoassay (LFI), and indirect hemagglutination test (IHA), while greatly reducing the detection time required (25 – 27), also suffer from similar drawbacks such as cross-reaction with nontarget bacteria which lead to misdiagnosis and incorrect antibiotic treatment (28, 29). Furthermore, these methods do not provide information on pathogen drug resistance, making it challenging to select appropriate antibiotics in a timely and accurate manner, which can lead to disease progression. As a result, there is a need for improved diagnostic methods that overcome these limitations and allow for rapid, sensitive, and accurate detection of melioidosis, including its drug resistance characteristics.

RNaseH-dependent PCR (rhPCR) amplification, which relies on the cleavage activity of thermostable RNase HII enzymes during hybridization to the complementary target sequence, offers several advantages that can enhance PCR (30). Firstly, the enzyme has minimal activity at low temperatures, allowing for a “heat-start PCR” without the need to modify the DNA polymerase. Secondly, the enzyme’s cleavage efficiency decreases in the presence of a mismatch near the RNA residue. Leveraging these properties can be designed for the target sequence, reducing the formation of primer dimers, improving specificity, enabling multiplex PCR with multiple primers, and facilitating the simultaneous detection of SNP (30). Lateral flow strip assay (LFSA) is a technique that combines colloidal gold particles, monoclonal antibody technology, and paper chromatography. LFSA holds great potential for nucleic acid analysis due to its visual detection, rapid response, ease of operation, and compatibility with portable cameras or smartphones. LFSA has already found applications in various fields, including protein analysis, small molecule detection, nucleic acid analysis, and exosome detection (31, 32).

In this study, we aimed to achieve rapid identification of *B. pseudomallei* and POCT for the detection of resistance mutation sites in the *penA* gene. To accomplish this, we employed rhPCR to amplify the specific house-keeping gene ORF2 of *B. pseudomallei* and P174L mutation site in *penA* known to be responsible for altered drug resistance. The amplification process was facilitated by the inclusion of universal primer probes and a simple temperature cycler reaction. Finally, the resulting products were differentiated based on the bands observed with LFSA. This platform demonstrates simplicity, user-friendliness, high sensitivity, specificity, and adaptability, making it a promising tool for POCT to detect other disease-related genes and SNPs, among other applications. By leveraging the advantages of this platform, it holds the potential for rapid and on-site detection of a wide range of disease-related genetic variations, providing valuable insights for clinical diagnosis and treatment decisions.

## MATERIALS AND METHODS

### Reagents and materials

Primers used in this study were synthesized, high performance liquid chromatography (HPLC) purified by Sangon Inc. (Shanghai, China). Deep Vent (exo-) DNA Polymerase (M0259S) and Deoxynucleotide (dNTP) Solution Mix (N0447S) were purchased from New England Biolabs (NEB, USA). EvaGreen (20× in Water) were purchased from Biotium (USA). Thermostable RNase-HII (A006S) was acquired from MAGIGEN Biotechnology Co. LTD. (Guangzhou, China). Capillary electrophoresis sequencing was conducted to confirm the allele sites by Sangon Inc. (Shanghai, China). Polymerase chain reaction (PCR) and quantitative real-time PCR (qPCR) were performed in Biometra TOne 96 and qTower3 Real-Time Detection System (Analytik Jena GmbH, Germany). LFSA assay was conducted using double-labeled nucleic acid test strips obtained from Genenode (Wuhan, China). For 12% native PAGE gel electrophoresis, an electrophoresis system (DYY-6C, LIUYI, China) was used, and gel images were captured using the Bio-Rad Imaging System (Bio-Rad, USA).

### Bacteria collection and genomic DNA extraction

*B. pseudomallei* strain HNBP001, referred to as Bp, was isolated from our laboratory (GenBank accession numbers CP038805 and CP038806). Thirty *B. pseudomallei* isolates, including 4 strains containing P174L mutation [referred to as Bp (P174L)], and 2 *B. cepacia* isolates (Bc) were obtained from clinical samples of patients admitted to the Second Affiliated Hospital of Hainan Medical University. Reference strains, including *Enterococcus faecalis*, *Staphylococcus aureus*, *Pseudomonas aeruginosa,* and *Escherichia coli*, used for specificity testing were obtained from ATCC (Table 1). Unless specified otherwise, bacteria were incubated at 37°C with oxygen in an incubator or shaken at 220 rpm in a shaker. Routine cultures were grown in Luria-Betani (LB) liquid or solid medium. Bacteria for drug susceptibility testing were grown in Mueller-Hinton (MH) liquid medium. All procedures involving live pathogens were conducted in a Biosafety Level 2 facility at the School of Tropical Medicine, Hainan Medical University. For rapid extraction of whole bacterial genomic DNA, the bacterial genome extraction kit (TIANGEN, China) was used following the heat lysis method instructions. The resulting supernatant was collected, and the DNA concentration was determined using a NanoDrop One/OneC Microvolume UV-Vis Spectrophotometer (Thermo Fisher Scientific, USA). Adjust the genomic DNA (gDNA) to the required concentration according to the experimental requirements; 1 µL supernatant was served as template for the latter PCR assays and sequencing.

**TABLE 1:** Strain information involved in this protocol

Strains	Sex/age	Source place	Specimen types	Symptom	Outcome	CAZ resistance
Bp No.1	Man/57	Dongfang	Blood	Pulmonary infection and sepsis	Recovered	Sensitive
Bp No.2	Man/58	Dongfang	Bronchoalveolar lavage fluid	Pulmonary infection	Recovered	Sensitive
Bp No.3	Man/31	Ledong	Joint fluid	Sepsis	Recovered	Sensitive
Bp No.4	Man/39	Qiongzhong	Blood	Sepsis	Recovered	Sensitive
Bp No.5	Man/46	Ledong	Blood	Sepsis	Recovered	Sensitive
Bp No.6	Man/60	Danzhou	Sputum	Sepsis	Recovered	Sensitive
Bp No.7	Man/51	Haikou	Sputum	Pulmonary infection and septic arthritis	Recovered	Sensitive
Bp No.8	Man/68	Sanya	Urine	Sepsis	Recovered	Sensitive
Bp No.9	Man/57	Haikou	Blood	Sepsis	Recovered	Sensitive
Bp No.10	Man/66	Dingan	Blood	Sepsis	Recovered	Sensitive
Bp No.11	Woman/30	Changjian	Blood	Pulmonary infection and sepsis	-^*a*^	Sensitive
Bp No.12	Man/80	Haikou	Blood	Pulmonary infection and sepsis	Died	Resistance
Bp No.13	Man/77	Dongfang	Blood	Pulmonary infection and septic arthritis	Died	Resistance
Bp No.14	Man/52	Dongfang	Bronchoalveolar lavage fluid	Pulmonary infection	Recovered	Sensitive
Bp No.15	Man/40	Wanning	Blood	Sepsis	Recovered	Sensitive
Bp No.16	Man/54	Danzhou	Blood	Pulmonary infection and sepsis	Recovered	Sensitive
Bp No.17	Man/49	Sanya	Bronchoalveolar lavage fluid	Pulmonary infection and sepsis	Recovered	Sensitive
Bp No.18	Man/36	Changjiang	Blood	Pulmonary infection and sepsis	-^a^	Resistance
Bp No.19	Man/59	Haikou	Blood	Sepsis	Recovered	Sensitive
Bp No.20	Woman/55	Wenchang	Joint fluid	Pulmonary infection and sepsis	Recovered	Sensitive
Bp No.21	Man/63	Dongfang	Bronchoalveolar lavage fluid	Pulmonary infection	Recovered	Sensitive
Bp No.22	Man/48	Changjiang	Blood	Sepsis	Recovered	Sensitive
Bp No.23	Man/64	Sanya	Bronchoalveolar lavage fluid	Pulmonary infection and sepsis	Recovered	Sensitive
Bp No.24	Man/48	−^a^	Blood	Sepsis	Recovered	Sensitive
Bp No.25	Man/66	Dongfang	Bronchoalveolar lavage fluid	Pulmonary infection and sepsis	Recovered	Sensitive
Bp No.26	−^a^	−^a^	−^a^	Pulmonary infection and sepsis	Recovered	Sensitive
Bp No.27	Man/56	Wenchang	Blood	Sepsis	Recovered	Sensitive
Bp No.28	Woman/11 months	Tunchang	Blood	Sepsis	−^*a*^	Sensitive
Bp No.29	−^a^	−^a^	−^a^	−	Recovered	Sensitive
Bp No.30	−^a^	−^a^	−^a^	Pulmonary infection and sepsis	Recovered	Resistance
Bc No.1	Man/57	Wanning	Bronchoalveolar lavage fluid	Chronic obstructive pulmonary disease	Recovered	Sensitive
Bc No.2	Woman/54	Haikou	Sputum	Pulmonary infection	Recovered	Sensitive
*E. faecalis*		ATCC29212				
*S. aureus*		ATCC25923				
*P. aeruginosa*		ATCC27853				
*E. coli*		ATCC25922				

^*a*^ No subsequent information was traced.

### Determination of the growth curves and antibiotic drug susceptibility

Freshly grown single colonies were selected and inoculated into 5 mL of LB liquid medium until reaching the log phase. The bacterial solution was then diluted to OD600 = 0.05. Two hundred microliters of bacterial solution was transferred to a 96-well plate and incubated at 37°C, 180 rpm for 24 hours, with the absorbance at OD 600 being measured continuously at 1-hour intervals. Each well should be repeated three times. These procedures were performed using aseptic techniques and were repeated independently on different dates for a total of three replicates. Strains requiring drug susceptibility testing in LB fresh media were grown in a 37°C incubator until the OD600 = 0.5. The Kirby-Bauer (K-B) test and microbroth dilution susceptibility test were performed according to the experimental method of each kit instruction.

Briefly, for the K-B test, use a sterile cotton swab to dip in bacterial liquid and squeeze out the excess bacterial fluid. Apply the entire MH surface with swab to ensure that the bacterial fluid is evenly applied. Use sterile tweezers to paste the drug sensitivity paper on the surface of the plate after the water was absorbed. The whole process should be completed within 15 minutes after bacterial inoculation. Then, the plate was reversed and inoculated at 37°C for 18–24 hours and the coil diameter was measured with a vernier caliper.

Microbroth dilution susceptibility test was performed according to the instruction with Zhuhai Deer non-fermentation bacteria drug susceptibility testing system (DL Biotech, China). Pure culture individual colonies were picked into 5 mL liquid LB and grown to log phase and adjusted to OD 600 = 0.1 with MH broth medium. Fifty milliliters of the above bacterial suspension was added to the matching MH broth medium tube; after completely mixing, 100 µL was added to each well of a 96-well plate corresponding to different antibiotics. In the process of dropping, pay attention to the suspension or replace the gun head in time to prevent cross-contamination between antibiotics. The reagent board was sealed and incubated at 37°C for 18–24 hours. The results and analysis reports were read using the microbial identification susceptibility analysis system.

### Primers, probe designing, and screening

To enable the simultaneous detection of the conserved sequence of *B. pseudomallei* and the P174L mutation site in *penA*, two pairs of primers were designed using the NCBI Primer-BLAST website, based on the whole genome of *B. pseudomallei* strain HNBP001 available in the NCBI database (GenBank accession numbers CP038805 and CP038806). The primer sequences are listed in Table 2. The product lengths were set in the range of 100 to 200 bp for rhPCR and real-time PCR assays and 400 to 500 bp for sequencing purposes. For LFSA biosensing strategy, primers used for ORF2 region analysis were labeled with biotin and FITC, while the primers used for the P174L mutation site analysis were labeled with digoxin and biotin. All sequences were evaluated using the NUPACK software tool (https://www.nupack.org/) to predicted the likelihood of secondary structure. Each rhPCR mixture consisted of 1 µL of extracted DNA (10 ng) in a total volume of 19 µL, including 2 µL of 10 × ThermoPol Reaction Buffer, 0.5U of Deep Vent (exo-) DNA Polymerase, 400 nM primers in total (with a ratio of blocking primers to universal primer probes of 1:10), 200 µM dNTP Solution, and 100 mU of RNase-HII. For quantitative fluorescence PCR, an additional 1 µL of 20 × EvaGreen was added for amplification and melt curve analysis. PCR was performed with the following parameters: an initial denaturation at 95°C for 5 minutes, followed by 45 cycles of 30 sec at 95°C and 45 sec at 65°C, with fluorescence signal collection at 65°C. For melt curve analysis, an additional warming process from 60°C to 95°C at a rate of 0.5°C per cycle was included, and the fluorescence was collected during this process. No-template controls were included in each run to account for contamination or potential amplification failures.

**TABLE 2:** Nucleotide sequences of primers and probes

Nucleotide		Sequence	Product size (bp)
ORF2	Primer-F	5′-GCACTCTGAGTCAGGCTAGACGTCTCTATACTGTCGAGCAATCG */rG/*GCGGT-C3 spacer-3’	155
	Primer-R	5′-CGAGGAGCTACTTCGGACTCCGTGCACACCGGTCAGTATC */rC/*CGTAT-C3 spacer-3’	
P174L	Primer-F	5′-GTACCGAGCGAGTTCATGAGTGAGCTGAACACGGCGCTGC */rT/*CGGCT-C3spacer-3’	189
	Primer-R	5′-CGAGGAGCTACTTCGGACTCCCGTCTTGTTGCCGAGCAT */rC/*CATGT-C3spacer −3’	
Probe 1	F	5′-FITC-GCACTCTGAGTCAGGCTAGA-3′	155
	R	5′-Biotin-CGAGGAGCTACTTCGGACTC-3′	
Probe 2	F	5′-Digoxin-GTACCGAGCGAGTTCATGAG-3′	189
	R	5′-Biotin-CGAGGAGCTACTTCGGACTC-3′	
Sequencing	F	5′-GCTTCAGTACAGCGACAACAC-3′	410
	R	5′-TAGACGATGAACACGATCGGC-3′	

### Lateral flow strip assay

The experiment was performed according to the instructions provided in double-labeled nucleic acid test strip kit from Genenode (Wuhan, China). Briefly, 10 µL of PCR-amplified products was absorbed into a new reaction tube and then diluted 10–20 times with the sample solution and fully mixed before testing. Next, 100 µL of diluted amplification products was dropwise added into the colloid gold detection hole. The test results were observed visually by examining the color development of the test strip within 3–10 minutes after the control line (C line) had developed color. The color intensity or presence of a line in the test region provided the basis for interpretation of the results. To document the results, the strip was photographed using a smartphone. After usage, the test strip should be placed in a sealed bag and disposed of appropriately.

### Data analysis

In order to determine the suitability of the established method for large-scale application, it is important to assess its accuracy, reproducibility, and stability. To evaluate the accuracy of detecting clinical samples, we tested 36 genomic gDNA samples and bacterial samples using the established method, and the results were compared with the gold standard obtained by sequencing. Each experiment was repeated three times to assess the stability of the experimental protocol. SPSS 20.0 statistical software was used to conduct the analyses. Pairwise comparisons were performed using the *t*-test and more than three comparisons using one-way ANOVA. *P* < 0.05 indicates a significant difference.

## RESULTS

### Ceftazidime-resistant strain identification

**Fig 1:**
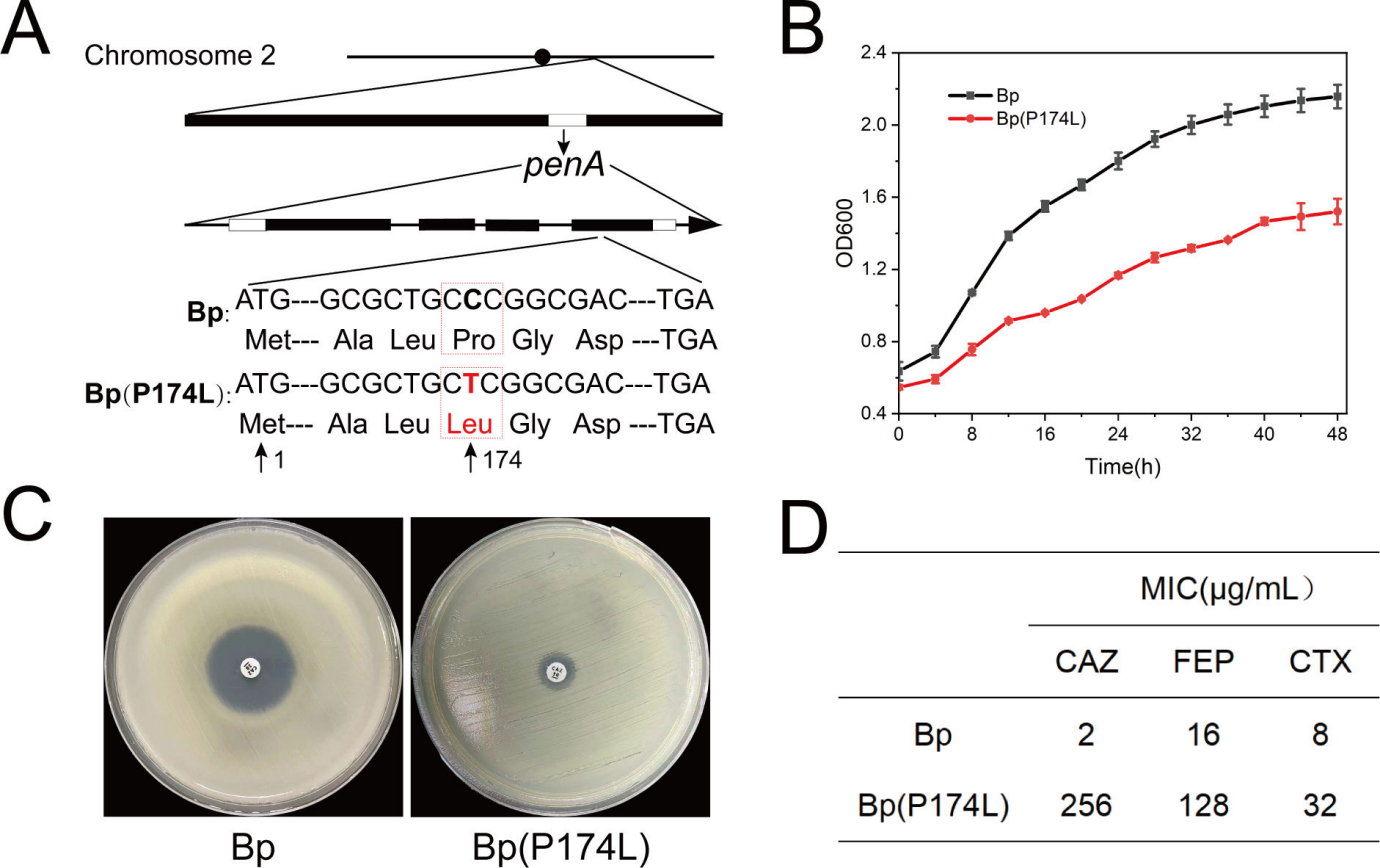
Characterization of CAZ-resistant Bp strain. Location of mutations in *penA* gene in the CAZ-resistant strain Bp (P174L) (**A**). Comparison of growth curves between Bp and Bp (P174L) (**B**). Determination of the antibiotic MIC between Bp and Bp (P174L) by K-B test (**C**) and microbroth dilution susceptibility test (**D**).

In this study, we conducted *penA* sequencing on a clinically isolated strain of *B. pseudomallei* that showed resistance to CAZ. The sequencing results revealed a *C* > *T* mutation in the conserved sequence of the *penA*, leading to an amino acid change from proline to leucine at position 174 (P174L), as depicted in Fig. 1A. This result was also consistent with the previous study which showed the same mutation type caused increased resistance to CAZ in *B. thailandensis* E264 strain reported by Yi, et al. (19). To investigate the impact of the P174L mutation on bacterial growth, we compared the growth curves of the resistant strain Bp(P174L) with the wild-type strain Bp. Turbidity measurements of the growth curves revealed no significant difference in the growth rate between the resistant and wild-type strains, as illustrated in Fig. 1B. Furthermore, we compared the CAZ drug susceptibility between Bp and Bp (P174L) using the K-B test and microbroth dilution susceptibility test. As shown in Fig. 1C and D, the CAZ-sensitive strain Bp exhibited a minimum inhibitory concentration (MIC) of 2 µg/mL, whereas the MIC of CAZ for Bp (P174L) increased to 256 µg/mL, indicating a 128-fold increase in resistance. Notably, this resistance phenotype remained stable during continuous passage culture. These findings demonstrate that the identified P174L mutation in the *penA* is associated with increased resistance to CAZ in *B. pseudomallei*, impacting bacterial growth and rendering the strain highly resistant to the antibiotic.

### Working principle

**Fig 2:**
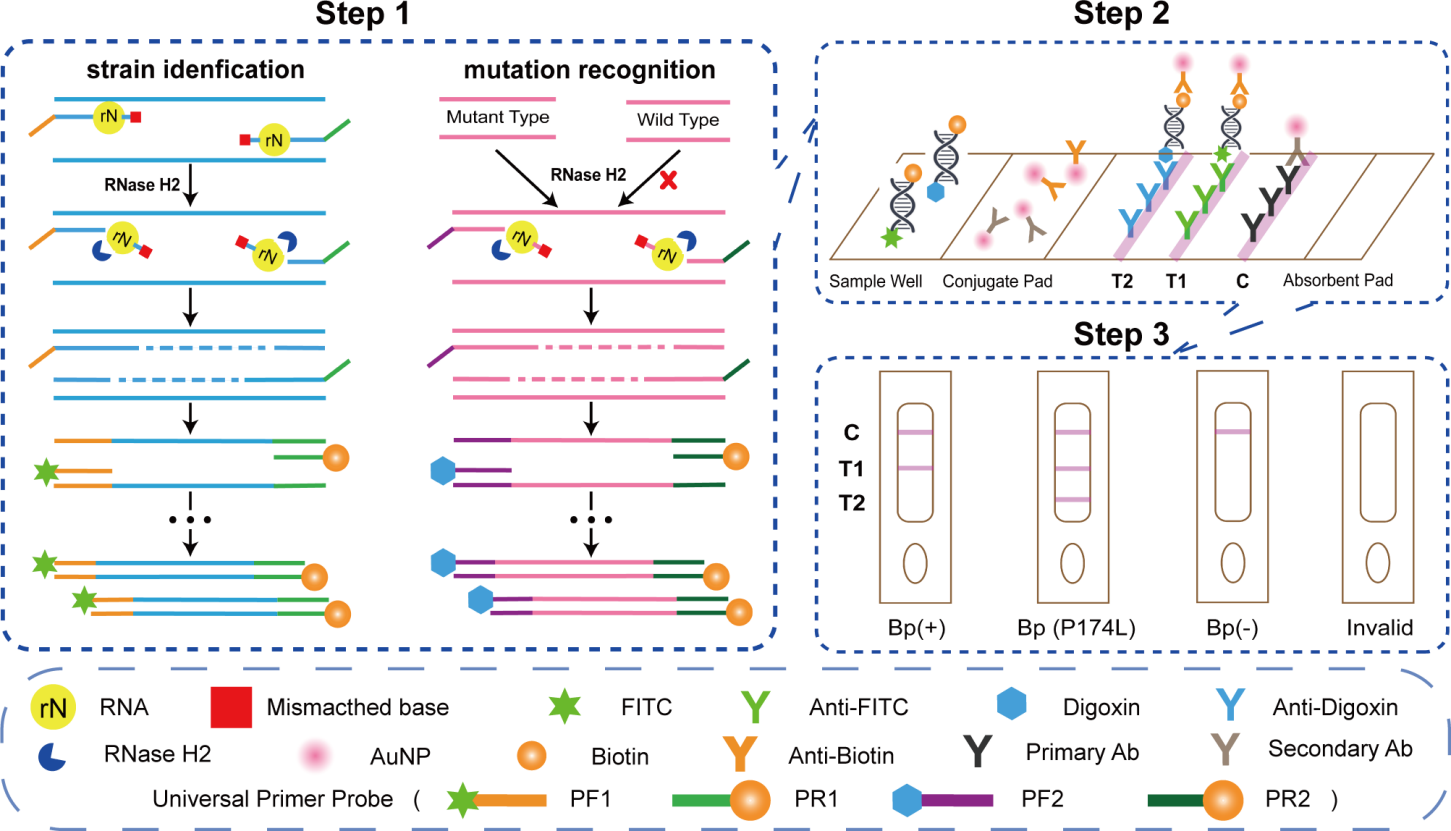
Scheme illustration of RNase-HII-dependent PCR combined with LFSA for strain identification and resistance mutation discrimination. Step 1 shows the concrete reaction process of rhPCR, step 2 shows the rationale of LFSA, and step 3 shows the result interpretation criteria.

The principle of the rhPCR system for visual detection of *B. pseudomallei* strains and the SNP of the *penA* is illustrated in Fig. 2. In step 1, PCR primers are designed with a single ribonucleotide residue near the 3′ terminus, which is complementary to the ORF2 region of *B. pseudomallei* and the P174L mutation site in the *penA*. The primers are unable to be extended by the DNA polymerase, and they form a substrate for RNase-HII. When the primer-template complex is present, the thermostable RNase-HII enzyme hydrolyzes the primer 5′ down to the RNA base, leaving a DNA oligonucleotide with a 3′-OH group capable of initiating DNA synthesis. The presence of *B. pseudomallei*-specific ORF2 or the P174L mutation allows for amplification to occur selectively. To enhance specificity, additional bases are added at the 3′ terminus, with the last base modified by a C3-spacer. The upstream and downstream primers also contain additional non-complementary sequences at their 5′ ends. Two pairs of universal probes are introduced into the system. One pair carries FITC and biotin tags, while the other pair carries digoxin and biotin tags. The sequences of these universal probes are consistent with the added part of the primer sequences. After the initial round of PCR amplification, the added sequences serve as templates for the universal probes, initiating subsequent PCR reactions. This generates PCR products with different tags. In step 2, the amplified products are used for LFSA. When the labeled double-stranded DNA carrying the biotin tag flows from the sample pad to the conjugate pad, the biotin antibody carrying gold nanoparticles (AuNPs) binds to the DNA. These complexes then flow to test line 1 (T1), where a fixed anti-FITC antibody captures the FITC tag, generating a red line to indicate the presence of *B. pseudomallei*. In the case of the digoxin/biotin double-labeled amplicon specific to the P174L genotype, the complexes flow to test line 2 (T2), where a fixed anti-digoxin antibody captures the digoxin tag, generating a red line. *B. pseudomallei* strains carrying the P174L mutation will produce red lines at both T1 and T2. A control line (C) is included to ensure the reliability of the test.

### Optimization of the rhPCR system

**Fig 3:**
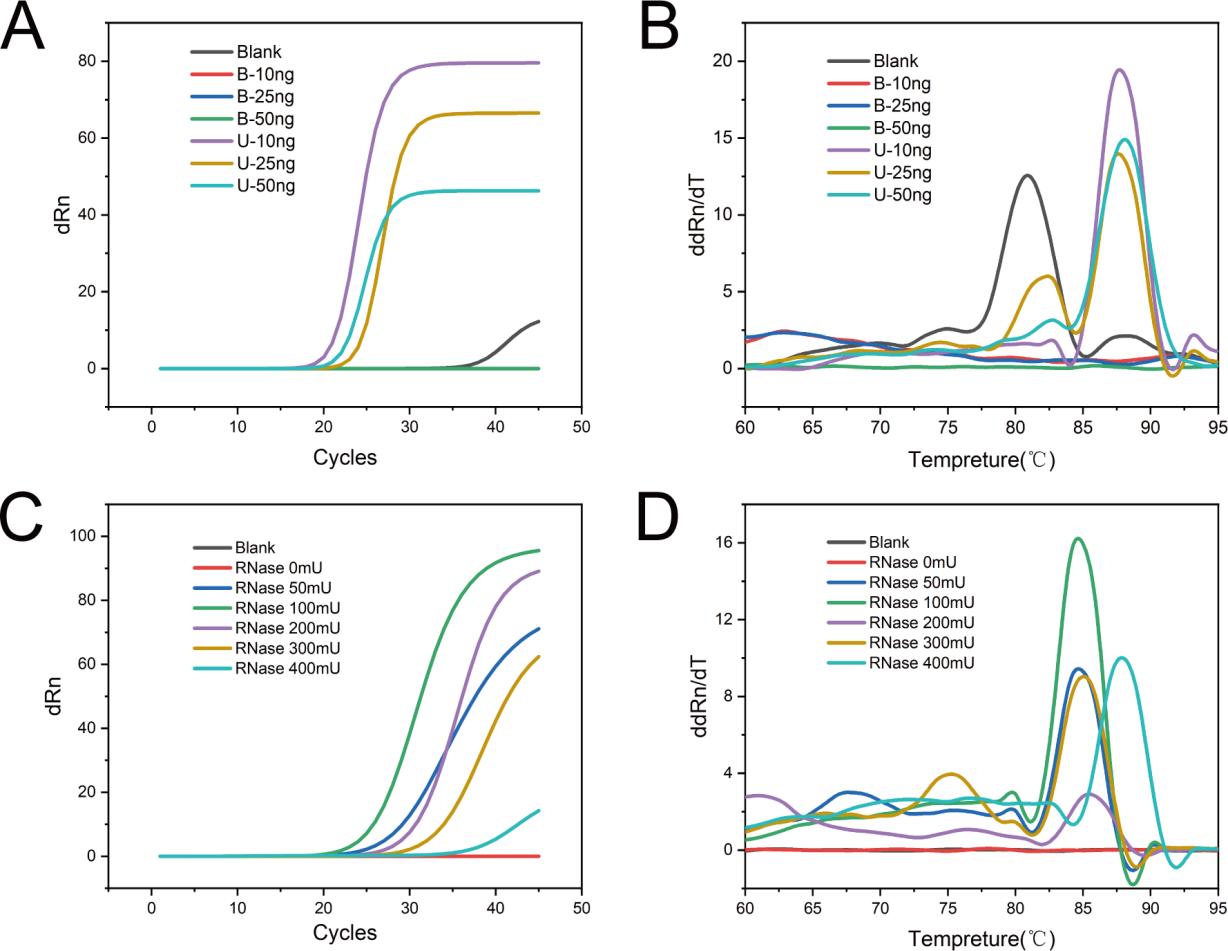
Optimization of the reaction conditions of rhPCR system. Analysis of the amplification (**A**) and melt curves (**B**) of the reactions at different template concentrations for ORF2. B represents the blocking primers, and U represents the unblocking common primers. Amplification (**C**) and melt curves (**D**) of the reactions at different RNase-HII concentrations for ORF2.

To optimize the template reaction concentration, a PCR reaction system was established using normal primers (unblocking primers) with the addition of the fluorescent dye EvaGreen. The PCR amplification curve and melt curve analyses were used to evaluate the performance. It was found that a template concentration of 10 ng yielded better Ct values and resulted in a single specific amplification product without any non-specific amplification (Fig. 3A and B; Fig. S1A). Similarly, the optimal amount of RNase-HII enzyme was determined using specific RNase-HII-dependent primers (blocking primers). The reaction performance was evaluated with varying amounts of RNase-HII, and it was observed that the best results were obtained when 100 mU of RNase-HII was added to the 20 µL reaction system (Fig. 3C and D; Fig. S1B). To determine the optimal annealing temperature for the rhPCR system, different annealing/extension temperatures (63°C, 65°C, and 67°C) were evaluated. The detailed RT-PCR data are presented in Fig. S2. Based on the results, it was determined that the system exhibited optimal amplification efficiency at an annealing temperature of 65°C. Therefore, this temperature was selected for the subsequent reactions. These optimization steps ensured that the PCR reactions using rhPCR with the designed primers and RNase-HII enzyme were performed under the most favorable conditions, maximizing amplification efficiency and specificity.

### Feasibility of the rhPCR-LFSA system

**Fig 4:**
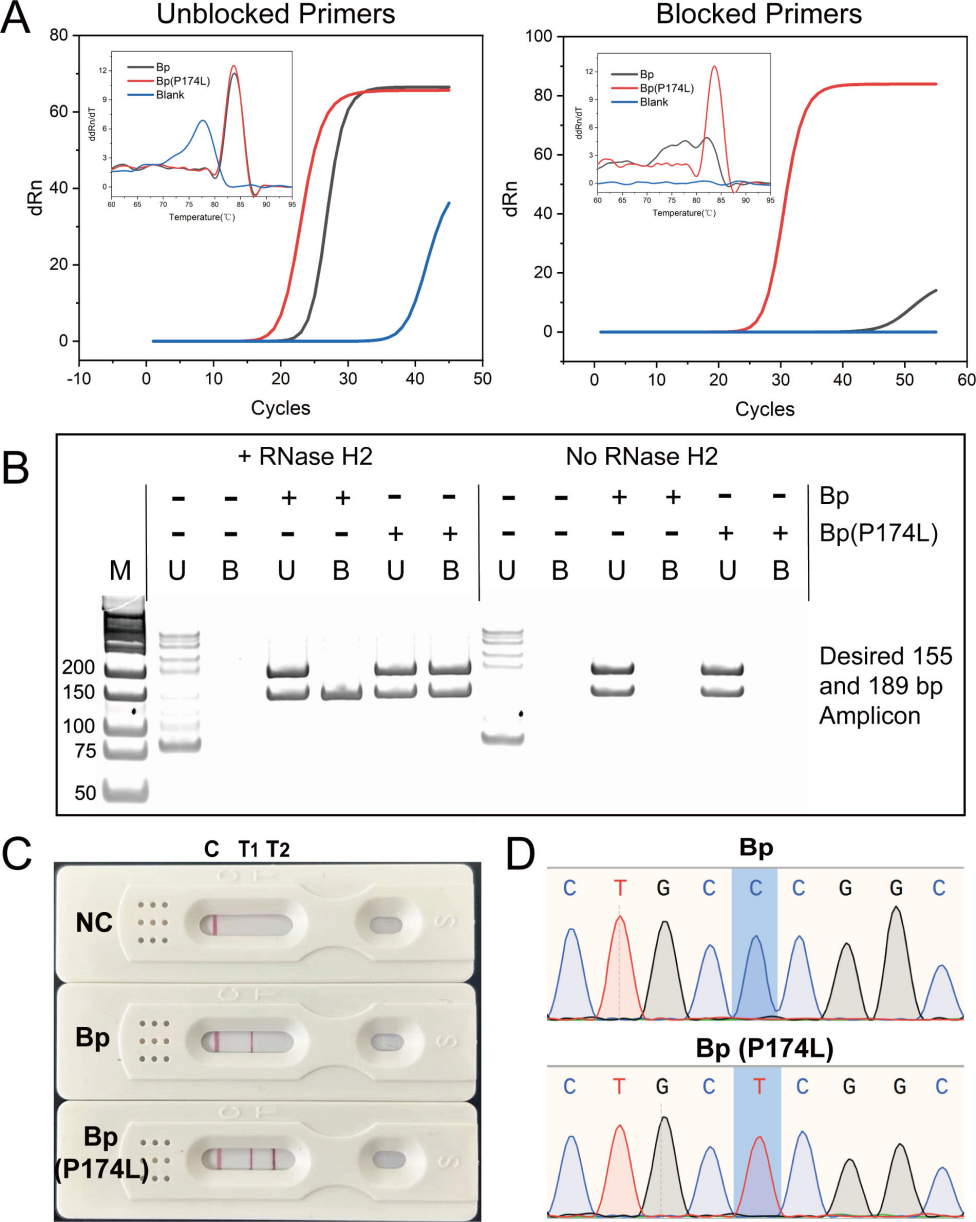
Feasibility analysis of the rhPCR-lateral flow system. Amplification curves in common (A, left) and blocked primer (A, right) reaction systems using different templates. Twelve percent native PAGE analysis of amplified products in complex nucleic acid environments under multiplex reaction conditions (**B**). U represents the unmodified common primers and B represents the blocked primers carrying RNA bases and C3-spacer group. LFSA results (**C**) and typical sequencing results (**D**) for the genotypes of Bp, Bp (P174L), and negative controls.

To evaluate the effectiveness of blocking primers in identifying mutant *B. pseudomallei*, PCR assays were performed using the P174L mutation as an example. It was observed that only the blocking primers produced specific PCR products in the Bp (P174L) group, while the wild-type Bp and blank control groups showed no amplification (Fig. 4A). In contrast, when using regular primers, amplification products were obtained in both the Bp and Bp (P174L) groups, making it difficult to identify the mutation site. Additionally, the control group showed non-specific products, leading to interference in detection. To further analyze the amplification products, a single tube containing the two reactions (ORF2 and P174L) was prepared and universal primers were added. The resulting complex nucleic acid environment was subjected to 12% native PAGE analysis. It was found that the use of unmodified primers produced multiple amplified species, including “primer dimer” products, which were independent of the input target and deviated from the expected amplicon sizes of 189 bp and 155 bp. In contrast, amplification using blocking cleavable primers was dependent on the presence of RNase-HII. The rhPCR format using cleavable primers produced the correct amplicon sizes (189 bp and 155 bp) when the Bp (P174L) target was present and a 155-bp amplicon when the Bp target was present. No amplified products were observed when the target was absent. The use of cleavable primers in the rhPCR system eliminated the formation of primer-dimer artifacts, resulting in more specific and accurate amplification (Fig. 4B). After PCR amplification, the products were subjected to LFSA. The LFSA results, as well as the typical sequencing results for the genotypes of Bp, Bp (P174L), and negative controls, are shown in Fig. 4 (C and D). LFSA was found to be a simple and easy-to-operate method that did not require expensive instruments, making it suitable for rapid and convenient detection. These results demonstrated the effectiveness of the rhPCR system combined with LFSA for the simultaneous detection of *B. pseudomallei* and the P174L mutation, highlighting its potential as a reliable and accessible tool for diagnostic applications.

### Clinical application of the rhPCR-LFSA system

**Fig 5:**
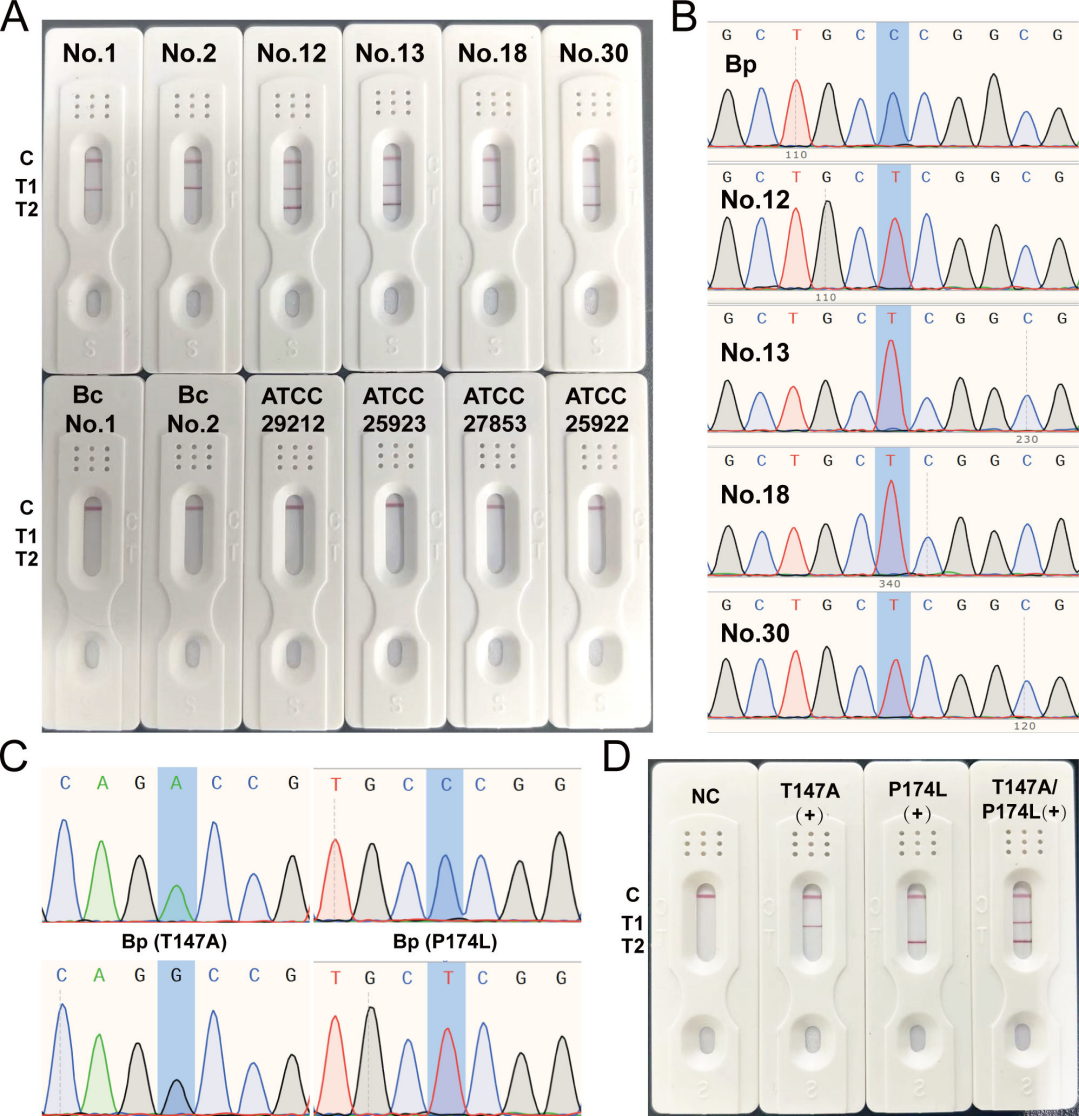
Clinical application of the rhPCR-LFSA system. Results of clinically derived Bp, Bc, and standard strain tests from ATCC (**A**). Sequencing results of the normal Bp strains and four strains carrying the P174L mutation (**B**). T147A alteration caused by A to G mutation at position 457 nucleotide of *penA* (**C**) and corresponding LFSA results (**D**).

To evaluate the clinical application of the system, a total of 32 culture isolates derived from hospitalized patients (30 *B. pseudomallei* isolates and 2 *B. cepacia* isolates were contained, of which 4 *B. pseudomallei* isolates carried P174L mutation) and four standard strains from ATCC were analyzed. The results showed that all clinically verified *B. pseudomallei* strains were detected and showed a positive signal on the T1 line of the LFSA strip. In contrast, *B. cepacia* strains and the other four reference strains from ATCC only showed bands in the control lines, with negative results in the test lines. Additionally, four strains were positive for the T2 test line, indicating the presence of the P174L mutation in these samples (Fig. 5A). To confirm the accuracy of the method, the P174L locus in these four strains was confirmed by sequencing. The sequencing results showed perfect agreement with the LFSA results, with a coincidence rate of 100% (Fig. 5B). Furthermore, a population genomic analysis of *B. pseudomallei* in Hainan Island, China, revealed a specific transition from A to G at position 457 nucleotide of *penA* (T147A) in approximately 60% of the Hainan *B. pseudomallei* genome (Fig. 5C) (17). To test the universality and transferability of the protocol, specific blocking primers targeting the T147A site carrying the universal probe 1 sequence were designed. The LFSA of the 30 *B. pseudomallei* isolates was performed after amplification using these primers. The results demonstrated the excellent universality of the protocol, enabling the simultaneous detection of two mutation sites in a single tube, with a coincidence rate of 100% compared with the sequencing results (Fig. 5D; Table 3; Fig. S4). These findings highlight the robustness and reliability of the developed system in clinical settings, providing a promising approach for the rapid and accurate detection of *B. pseudomallei* and its associated drug resistance mutations.

**TABLE 3:** The comparative results for the LFSA with DNA sequencing

Genotype	Number of test		Coincidence
	LFSA	DNA sequencing	
P174L (+)	4 (30)	4	100%
T147A (+)	11 (30)	11	100%
P174L / T147A (+)	2 (30)	2	100%

## DISCUSSION

Melioidosis, a severe infectious disease caused by *B. pseudomallei*, remains a global concern due to its high morbidity and mortality rates. While the disease was initially identified in 1912, its significance surged following the Vietnam War when melioidosis gained attention due to its substantial impact on the US military (4). The disease’s endemicity in northern Australia and Southeast Asia, along with sporadic cases in North America, has highlighted the challenges in its diagnosis and management, compounded by its diverse clinical manifestations and high antibiotic resistance (5, 33 – 36). The rising incidence in China, particularly since the initiation of the National Health Surveillance Program, underscores the need for improved diagnostics and treatment strategies, especially in the face of emerging drug resistance.

In this study, we focused on addressing the diagnostic challenges of melioidosis and the emergence of drug resistance. The current gold standard relies on bacterial cultures, which can be time-consuming. Molecular techniques, such as PCR targeting specific genes like the type III secretion system (TTS1) gene cluster, have shown promise in identifying *B. pseudomallei* strains. Notably, the resistance of *B. pseudomallei* strains to key antibiotics like CAZ, a cornerstone of treatment, is becoming a concerning issue. Our study delves into a fatal case involving a penA alteration resulting in high CAZ resistance. Through whole-genome sequencing (WGS) analysis, we identified a P174L substitution in penA associated with a significant increase in minimum inhibitory concentration. This highlights the critical need for a rapid diagnostic method capable of identifying both the presence of *B. pseudomallei* and specific drug resistance mutations to inform targeted treatment decisions.

Our contribution to this challenge is the development of a PCR protocol utilizing thermostable RNase-HII enzymes. This protocol aims to expedite *B. pseudomallei* identification and improve diagnostic reliability, especially in endemic regions. By incorporating the identification of the P174L mutation, associated with CAZ resistance, we enhance the diagnostic accuracy of our system. Specificity is achieved through blocking primers’ design and the cleavage activity of thermostable RNase-HII enzymes. The compatibility of these enzymes with DNA polymerase and their reduced cleavage efficiency in the presence of mismatches near RNA residues allow for multiplex PCR and mutation detection.

Importantly, in resource-limited settings where technical expertise and equipment are scarce, our PCR protocol integrated with lateral flow strip assay presents a practical solution. LFSA, utilizing colloidal gold particles and paper chromatography, offers visual detection and simplicity, making it suitable for point-of-care testing (POCT) (37, 38). We incorporated LFSA into our PCR system, introducing universal detection probes consistent with blocking primer sequences. These probes, labeled for LFSA detection, generate red bands on the strip upon flow, enabling visual identification without the need for expensive equipment. This user-friendly approach is well suited for primary healthcare settings.

Our study showcases successful detection of T147A and P174L sites in *B. pseudomallei* strains using LFSA, with results matching sequencing outcomes. In summary, the integration of LFSA into our PCR protocol presents a promising avenue for improved diagnosis and management of melioidosis in resource-constrained regions. This approach’s simplicity, speed (2.5–3 hours), and adaptability for various strains and drug resistance sites hold significant potential. However, we acknowledge the current need for DNA extraction, a limitation we aim to address in future studies to enhance the protocol’s accuracy. Moreover, expanding the sample size will contribute to assessing the protocol’s specificity and sensitivity more comprehensively.

## SUPPLEMENTAL MATERIAL

Supplemental figures and tables spectrum.01125-23-s0001.docx Table S1 and Fig. S1 to S4.

ASM does not own the copyrights to Supplemental Material that may be linked to, or accessed through, an article. The authors have granted ASM a non-exclusive, world-wide license to publish the Supplemental Material files. Please contact the corresponding author directly for reuse.
