# Supplementary material for: Synchronous detection of Burkholderia pseudomallei and its ceftazidime resistance mutation based on RNase-HII hydrolysis combined with lateral flow strip assay
Source: Microbiol Spectr. 2023 Oct 10;11(6):e01125-23. doi: 10.1128/spectrum.01125-23 (PMC10714834; doi:10.1128/spectrum.01125-23)
Supplement: Supplemental figures and tables — Table S1 and Fig. S1 to S4. [file spectrum.01125-23-s0001.docx]

**Title**

**Synchronous detection of *Burkholderia pseudomallei* and its ceftazidime-resistance mutation based on RNase-HII hydrolysis combined with lateral flow strip assay**

Yao Juan,^a,b,1^ Zhang Zhang,^a,c,1^ Tian Shen,^a^ Luo Nini,^a^ Tan Jun,^a^ Zhang Yue,^a^ Gu Shuo,^c,*^ and Xia Qianfeng^a,*^

^a^Key Laboratory of Tropical Translational Medicine of Ministry of Education, NHC Key Laboratory of Tropical Disease Control, School of Tropical Medicine, Hainan Medical University, Haikou, Hainan 571199, PR China.

^b^Nanobiosensing and Microfluidic Point-of-Care Testing Key Laboratory of LuZhou, Luzhou, Sichuan 646000, PR China.

^c^Department of Neurosurgery, Neurology Center, The First Affiliated Hospital of Hainan Medical University, Haikou, Hainan 571199, PR China.

1. mail: gushuo007@hainmc.edu.cn

xiaqianfeng@hainmc.edu.cn

**Supplementary data**

**Table S1**

Nucleotide sequences of primers and probes

| Nucleotide | | Sequence | Product size  (bp) |
| --- | --- | --- | --- |
| ORF2  (normal primers) | Primer-F | 5’-CGTCTCTATACTGTCGAGCAATCG-3’ | 115 |
|  | Primer-R | 5’-CGTGCACACCGGTCAGTATC-3’ |  |
| P174L  (normal primers) | Primer-F | 5’-TGAGCTGAACACGGCGCTGC-3’ | 149 |
|  | Primer-R | 5’-CCGTCTTGTTGCCGAGCAT-3’ |  |
| T147A | Primer-F | 5’-GCACTCTGAGTCAGGCTAGAATCGCGCTGCTCGGCGGGCCGCAG***/rG/***CCGTT-C3 spacer-3’ | 174 |
|  | Primer-R | 5’-CGAGGAGCTACTTCGGACTCTCGTCGTATCGCGCTCGT***/rC/***GCCT-C3 spacer-3’ |  |


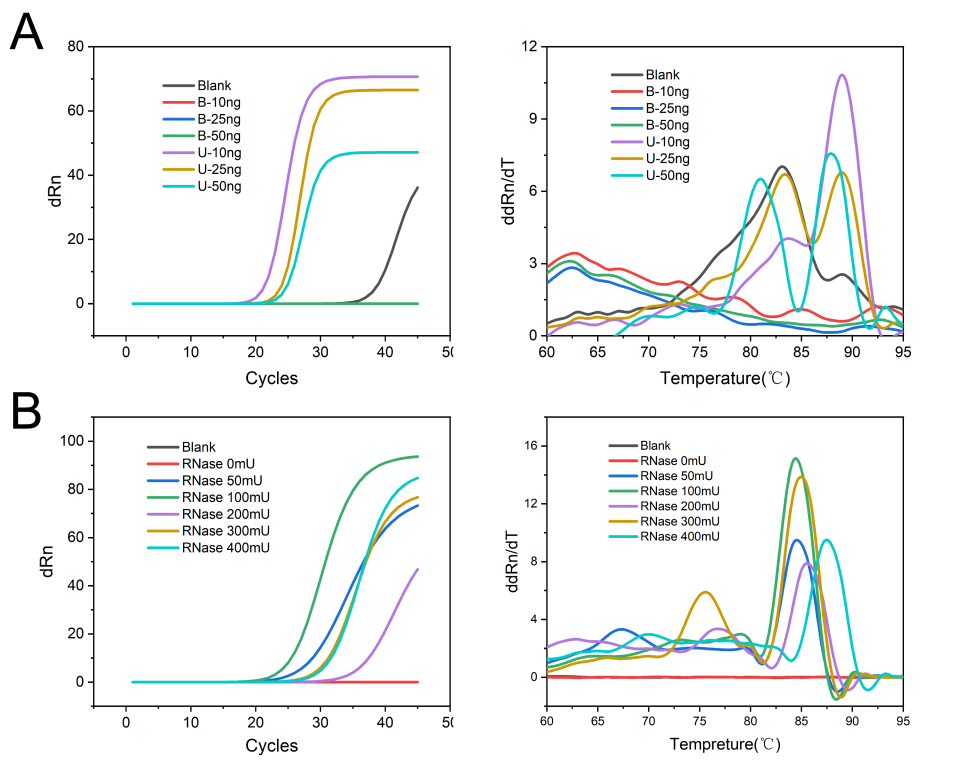


**FIG S1** Optimization of the reaction conditions of rhPCR system. Analysis of the amplification (**A**) and melt curves (**B**) of the reactions at different template concentration for P174L. **B** represents the blocking primers, and **U** represents the unblocking common primers. Amplification (**C**) and melt curves (**D**) of the reactions at different RNase-HII concentrations for P174L.


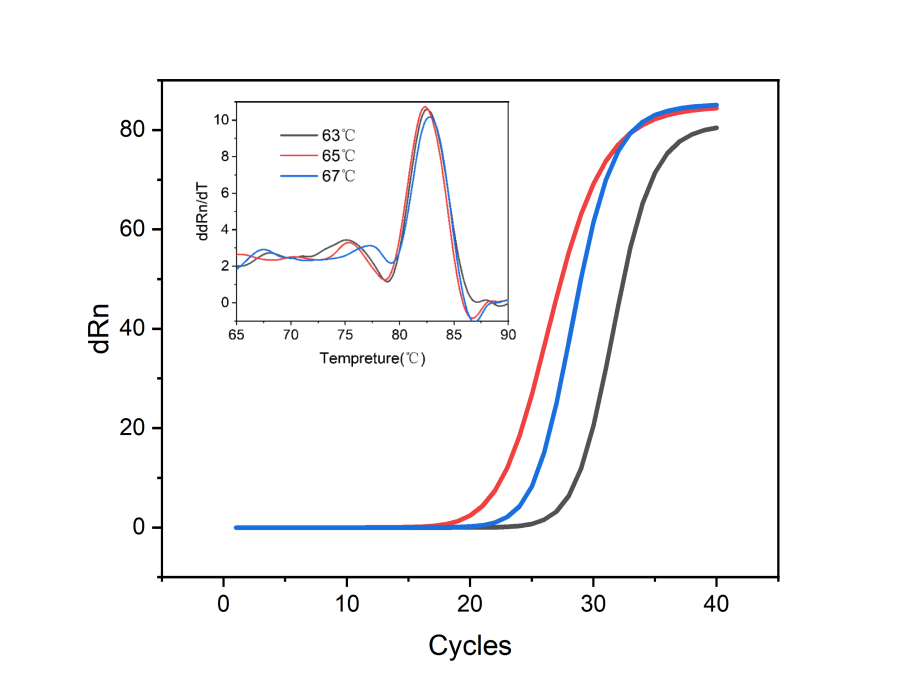


**FIG S2** Optimization of the annealing temperature of rhPCR system.


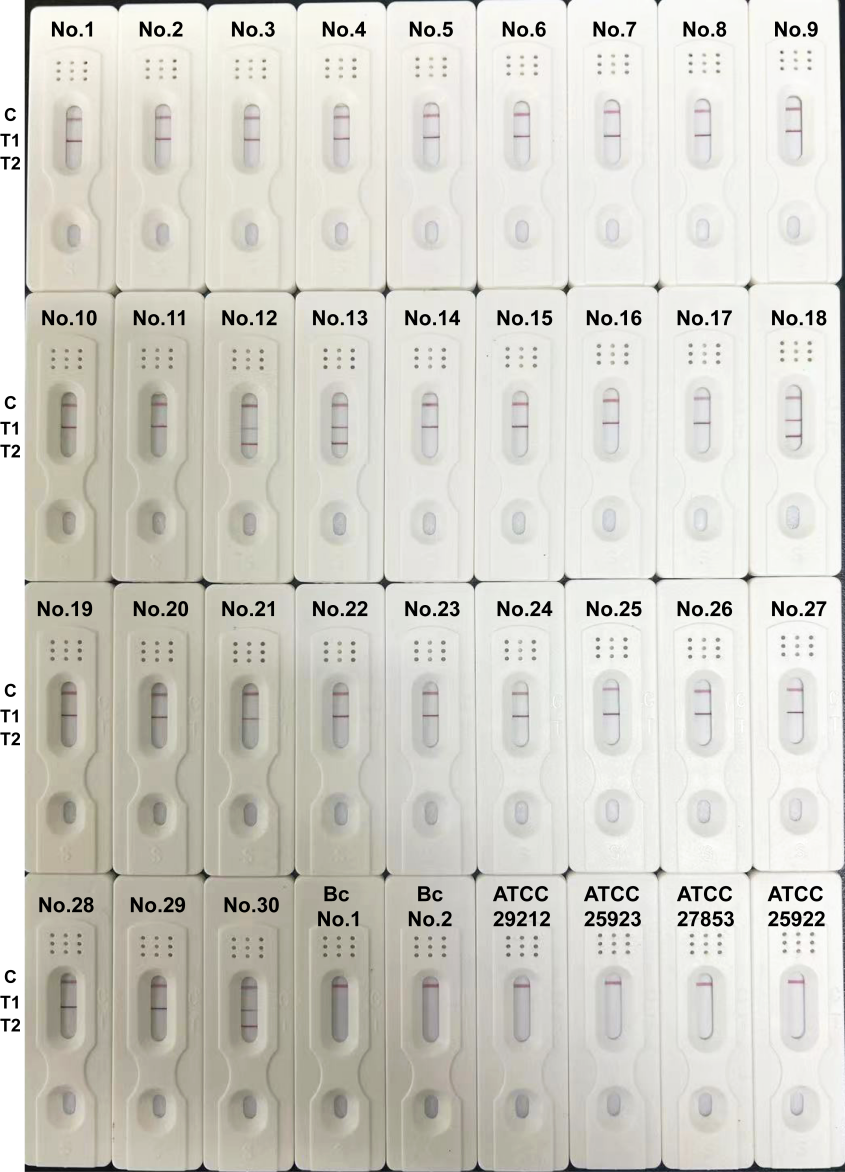


**FIG S3** LFSA results of 30 clinically isolated *B. Pseudomallei* specimens (No. 1-30), 2 clinically isolated *B. cepacia* specimens (Bc No. 1-2), and 4 reference strains derived from **ATCC** in Bp identification and P174L recognition system. T1 line represents *B. Pseudomallei* (+) and T2 line represents P174L (+).


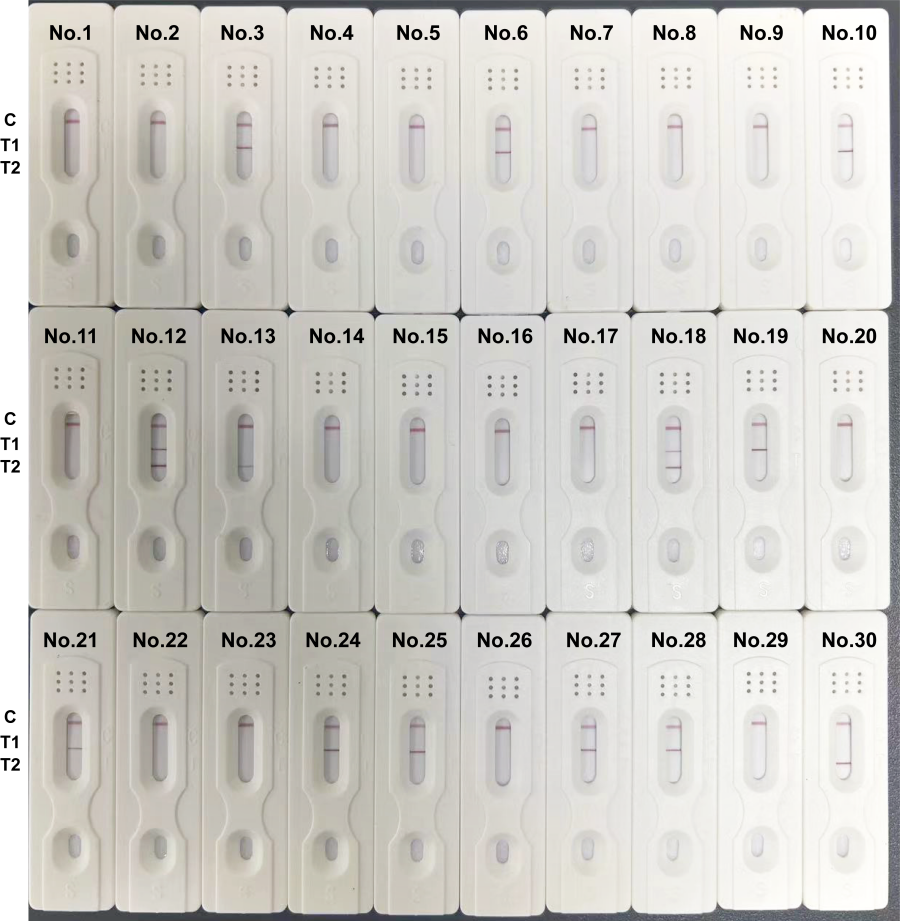


**FIG S4** LFSA results of 30 clinically isolated Bp specimens (No. 1-30) in simultaneously identification of T147A and P174L system. T1 line represents T147A (+) and T2 line represents P174L (+).
